# Delayed primary intention with full-thickness skin graft in distal interphalangeal injury: A rare case report

**DOI:** 10.1016/j.ijscr.2023.108155

**Published:** 2023-04-11

**Authors:** Alia Adelina Dina Soraya, Monica Bellynda, Achmad Luthfi Tiflani, Galuh Aretnaningtyas, Kristanto Yuli Yarso

**Affiliations:** a Resident of General Surgery, Sebelas Maret University, Moewardi Hospital, Surakarta, Indonesia; b Plastic and Reconstructive Surgery Division, Department of Surgery, Sebelas Maret University, Moewardi Hospital, Surakarta, Indonesia; c Oncology Surgery Division, Department of Surgery, Sebelas Maret University, Moewardi Hospital, Surakarta, Indonesia

**Keywords:** Full-thickness skin grafting, Skin graft, Fingertip injury, Case report

## Abstract

**Introduction and importance:**

The fingertip injury is the most common in the hand area. In this regard, skin grafting can be considered to preserve sensation, due to its functional importance, particularly for distal tip injuries. A full-thickness skin graft (FTSG) results in excellent function after engraftment and should be considered in reconstructing functionally and aesthetically important areas. Moreover, a thorough understanding of FTSG is required for a surgeon to have an excellent outcome.

**Case presentation:**

A 38-year-old man had the third fingertip injury of his right hand after being crushed by a mill. Physical examination revealed exposed bone distal to DIP, with intact periosteum and nail plate, negative active bleeding, and negative contaminants. There was no tendon or soft tissue left above the periosteum. In addition, an X-ray of the right manus revealed no fracture. The wound was applied with hydrogel and petroleum gauze to maintain hydration. A wound toilet was performed, followed by the closure of the wound with full-thickness skin grafting (FTSG). Follow-up was done in the first week and the fourth week after the procedure, as they showed good aesthetic results with satisfactory function. The sensory recovery showed normal result for touch and vibration. Meanwhile, sharp pain and warmth object sensation were minimally diminished.

**Clinical discussion:**

A literature review concludes that FTSGs are generally unreliable in cases with over poorly vascularized beds, and FTSG will only work with no serious blood supply issues. Therefore, severe fingertip injury was reconstructed by the graft.

**Conclusion:**

This procedure showed excellent graft survival with no additional surgical injury of the normal finger, satisfactory functional and aesthetic outcomes, and no need for secondary debulking procedures. Potential disadvantages consisted of insufficient volume of soft tissue and graft hyperpigmentation. However, delayed primary wound closure by FTSG may be an option for treating full-thickness finger defects with bone or tendon exposure.

## Highlights

• The fingertip injury is the most common in the hand area. In this regard, skin grafting is can be performed to cover skin defects. However, FTSG are not a commonly used option for this district, which may however benefit some patients in select cases where more complex options are not warranted.
• A full-thickness skin graft (FTSG) results in excellent function after engraftment and should be considered in reconstructing functionally and aesthetically important areas.
• We performed FTSG on 38-year-old man had the third fingertip injury of his right hand after being crushed by a mill.
• It revealed exposed bone distal to DIP, with intact periosteum and nail plate, negative active bleeding, and negative contaminants. There was no tendon or soft tissue left above the periosteum. In addition, an X-ray of the right manus revealed no fracture.
• This procedure showed excellent graft survival with no additional surgical injury of the normal finger, satisfactory functional and aesthetic outcomes, and no need for secondary debulking procedures.

## 1 Introduction

The fingertip is part of the digit distal to the insertion of the extensor and flexor tendons on the distal phalanx [1]. The distal part of the finger is composed of the distal phalanx, the nail bed soft tissue, and the nail edge. On the other hand, fingertip injuries can involve skin and pulp loss with or without exposed bone [2]. Generally, various types of flaps, including local, regional, and free flaps, have been used to cover small or moderate size full-thickness defects of the fingers [3]. In this study, written informed consent has been taken from the patient to publish this writing and images. This case aims to report the favorable outcome of delayed primary intention with FTSG for fingertip injury. Further, this case is written based on Consensus Surgical CAse REport (SCARE) Guidelines [4].

## 2 Presentation of case

**Fig. 1:**
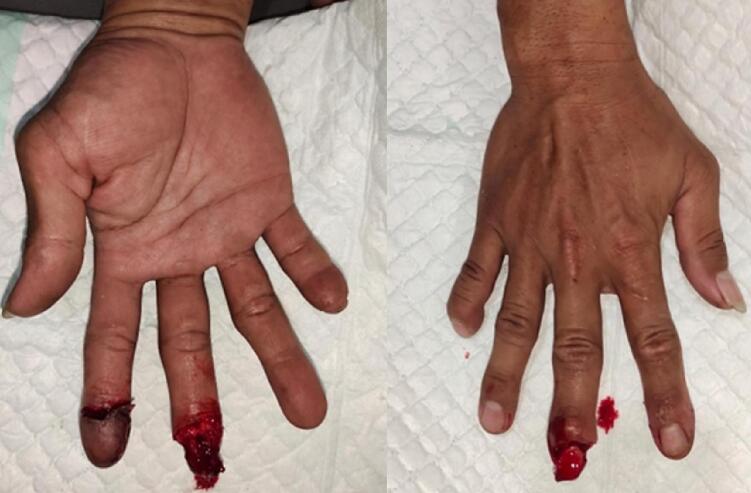
Preoperative view, before the full-thickness skin grafting application.

This study presents a case of a 38-year-old Javanese man presented with the third fingertip injury of his right hand after being hit by a mill. Physical examination revealed exposed bone distal to DIP, with intact periosteum and nail plate, negative active bleeding, and negative contaminants (Fig. 1).

**Fig. 2:**
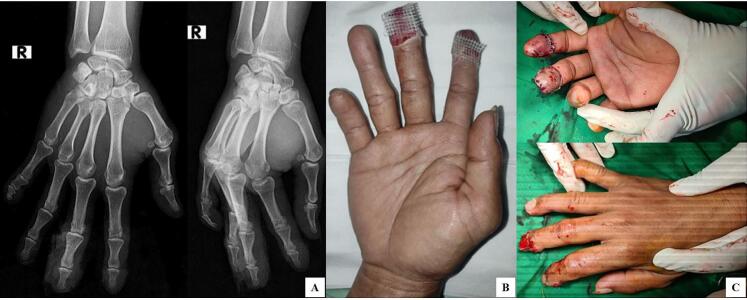
(A) X-ray examination of the right manus showed no fracture; (B) wound dressing; (C) postoperative view after FTSG application.

**Fig. 3:**
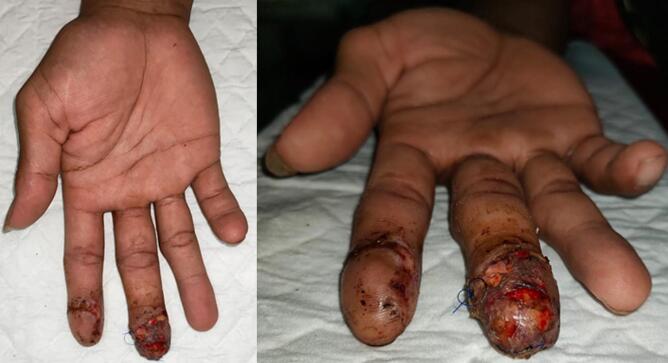
First-week follow-up.

**Fig. 4:**
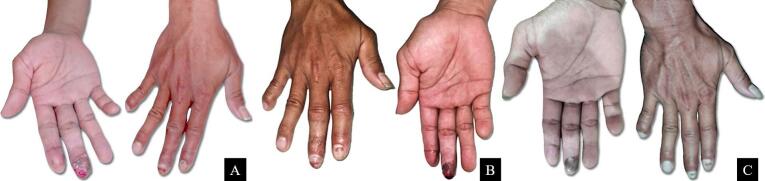
(A) Fourth-week follow-up, the patient was unable to identify sharp pain and temperature sensation; (B) sixth-week follow-up, he started to achieve dull pain and minimum warmth sensation; (C) third-month follow-up, several sensation such as touch and vibration were fully-recovered, however, sharp pain and warmth sensation were minimally-recovered.

A right manus x-ray was performed, resulting in no fracture (Fig. 2-A). The wound was covered with hydrogel and petroleum gauze to maintain hydration for two days (Fig. 2-B). After five days, granulation formation was found in fingertip shape. The wounds were irrigated thoroughly as devitalized tissue was debrided by the wound toilet. The skin graft was harvested from the groin area since it has different areas from traumatic areas and minimizes scarring in the future. The graft was sutured at the edge to intact skin utilizing a non-absorbable suture and then sutured to the nail plate as the patient's ingrowth nail was sutured (Fig. 2-C). The wound was dressed with petroleum gauze and then immobilized with spalk for two weeks. By then, follow-up was done in the first week (Fig. 3), the fourth week (Fig. 4-A), the sixth week (Fig. 4-B), and the twelfth week after the procedure (Fig. 4-C).

The favorable sensory recovery results of fingertip graft were obtained. The patient could identify touch and vibration sensation from cotton wool and 128 Hz tuning fork, respectively. However, sharp pain and warmth object sensory function were minimally diminished compared to the opposite fingers.

## 3 Clinical discussion

Fingertip injury is usually managed by secondary intention if the wound is less than 1 cm or by primary intention with flap or graft if the wound is greater than or equal to 1 cm. The primary intention with the graft will lead to poor aesthetic results and function. Hence, the flap is done to the nail deformity. When the wound is contaminated and left open due to infection prevention, its closure is performed after a few days. This condition is named delayed primary healing. The edges cannot be approximated when the tissue loss has been more extensive. Alternatively, the wound must be left open because of sepsis, and then the reparative process would be prolonged due to extensive granulation tissue that would fill the defect. This process is termed closure by secondary intention, where huge defects can heal in this process; however, the result is often less aesthetic than closed primary intention [5].

Concerning this, patients should be reminded that the donor vascular supply remains fragile for several weeks postoperatively. For this reason, showering with water directly on the area and excessive activity should be avoided for the next one to two weeks. In addition, FTSG complications can be classified into short-term problems and long-term functional and cosmetic problems.

Short-term problems can occur, such as infection, hematoma, seroma, and excessive displacement of the donor on the surface of the recipient. Although postoperative infection is rare, it is essential to carefully manage intraoperative tissues and minimize electrocautery-induced tissue necrosis. Prophylactic antibiotics for *Staphylococcus* and *Streptococcus* may be helpful, especially in patients with immunosuppression, diabetes mellitus, or long intraoperative times [6]. Moreover, the hematoma and seroma can be avoided by careful intraoperative hemostasis, pressure dressings, and postoperative care. Patients are recommended to avoid aspirin for ten days before surgery, NSAIDs five days before surgery, and alcohol two days before surgery and two days after surgery. Patients are also advised not to do strenuous activities to help inhibit donor movement and minimize disruption of blood supply to the donor from the recipient [7].

Furthermore, long-term complications for cosmetic and functional problems can occur, so it is necessary to educate that healing results can take months to achieve natural results. Make-up can be worn after three to four weeks after the procedure. In addition, it should be noted that the FTSG site is often depressed during the first two to four weeks. Meanwhile, spot dermabrasion can be performed after six weeks to six months to correct the difference in elevation between the graft site and the surrounding skin and improve color and texture match. Topical hydroquinone and/or tretinoin may also be useful in treating donor site hyperpigmentation [7].

Patients with finger injury that have not exposed the bone or tendon and have less than 2-cm skin loss (not 1 cm) are deemed not viable for surgery and are better off receiving secondary healing, which has been shown to improve sensate nerve recovery compared to skin grafting [8]. Furthermore, an algorithmic approach would have recommended homodigital, neurovascular island or thenar flap [9]. However, we decided to proceed differently as groin area has thick skin layer thus the patient could use his finger tip without limitation in future.

In this case, a full-thickness skin graft (FTSG) results in excellent function after engraftment and should be considered; however, FTSGs are generally unreliable in cases with over poorly vascularized beds. FTSG will work with no serious blood supply issues if only the healthy peripheral wound bed is wider than the avascular bone or tendon area. It should also be noted that the center of the graft can survive over a bone or tendon area through the bridging phenomenon [3]. In this regard, skin grafting is the gold standard for covering skin defects. Full-thickness skin grafts remove the entire epidermis and dermis, including adnexal structures such as hair follicles and sweat glands. Surgical principles of full-thickness skin grafting comprise wound bed preparation by tangential excision of granulation tissue and marginal de-epithelization of the normal skin, followed by coverage of full-thickness skin graft with preservation of the subdermal plexus on the central area and partial excision of the peripheral deep dermis of the graft [3], [6]. Nevertheless, this procedure is not applicable in the avascular area, such as bone, tendon or nerve without periosteum, perichondrium, and perineum [10].

## 4 Conclusion

While short-term and long-term complications might occur, delayed primary intention with FTSG demonstrated excellent graft survival and satisfactory functional and aesthetic outcomes with no debulking procedures. An overall understanding of FTSG includes indications, contraindications, preoperative evaluation, selection of donor sites, procedure techniques, postoperative care, and postoperative complications, which might help enhance the outcomes of this procedure. In short, FTSG may be considered for covering full-thickness finger defects with bone or tendon exposure.

## Source of support

This article is supported by Oncology Surgery Division, Department of Surgery, Sebelas Maret University, Moewardi Hospital, Surakarta, Indonesia.

## Ethical approval

This case is approved by Health Research Ethics Committee of Dr. Moewardi General Hospital (No. 1.454/XI/HREC/2022). Written informed consent was obtained from the patient for publication of this case report and accompanying images. A copy of the written consent is available for review by the Editor-in-Chief of this journal on request.

## Funding

The authors declared that this study has received no financial support.

## Guarantor

Kristanto Yuli Yarso.

## Research registration number

N/A.

## CRediT authorship contribution statement

All authors contributed equally to this manuscript. All authors read and approved the final manuscript. Galuh Aretnaningtyas and Kristanto Yuli Yarso: Chief of surgical procedure, conceptualization, reviewing, editing, supervision and validation of manuscript. Alia Adelina Dina Soraya, Monica Bellynda, Achmad Luthfi Tiflani: All clinical-laboratory examination, surgical procedure, writing-original initial draft and final manuscript.

## Conflict of interest

The authors declare no conflict of interest.
